# Supplementary material for: Enhancing fairness in disease prediction by optimizing multiple domain adversarial networks
Source: PLOS Digit Health. 2025 May 30;4(5):e0000830. doi: 10.1371/journal.pdig.0000830 (PMC12124548; doi:10.1371/journal.pdig.0000830)
Supplement: S1 Table — For the ADNI dataset, the majority group corresponds to individuals over 78 years old, with more than 18 years of education, and are right-handed. (PDF) [file pdig.0000830.s001.pdf]

Class	Age Groups		Educated Years		Handedness	
	<78 years	≥78 years	<16 years	≥16 years	Right-Handed	Left-Handed
NC	230 (65.7%)	120 (34.3%)	206 (58.9%)	144 (41.1%)	340 (97.1%)	10 (2.9%)
MCI	107 (70.4%)	45 (29.6%)	125 (82.2%)	27 (17.8%)	130 (85.5%)	22 (14.5%)
AD	113 (67.3%)	55 (32.7%)	139 (82.7%)	29 (17.3%)	150 (89.3%)	18 (10.7%)
