# Supplementary material for: Enhancing fairness in disease prediction by optimizing multiple domain adversarial networks
Source: PLOS Digit Health. 2025 May 30;4(5):e0000830. doi: 10.1371/journal.pdig.0000830 (PMC12124548; doi:10.1371/journal.pdig.0000830)
Supplement: S2 Table — For the Autism dataset, the majority group corresponds to the right-handedness and male subpopulation. (PDF) [file pdig.0000830.s002.pdf]

| Class | Sex | | Handedness | |
|---|---|---|---|---|
| | Male | Female | Right-Handed | Left-Handed |
| ASD | 56 (50.9%) | 54 (49.1%) | 104 (94.5%) | 6 (5.5%) |
| TC | 86 (84.3%) | 16 (15.7%) | 86 (84.3%) | 16 (15.7%) |
