# Supplementary material for: Enhancing fairness in disease prediction by optimizing multiple domain adversarial networks
Source: PLOS Digit Health. 2025 May 30;4(5):e0000830. doi: 10.1371/journal.pdig.0000830 (PMC12124548; doi:10.1371/journal.pdig.0000830)
Supplement: Attachment — Submitted filename: Plos___Rebuttal__new_.pdf [file pdig.0000830.s003.pdf]

Dear Editors,

Thank you for giving us the opportunity to submit a revised version of our manuscript titled "Enhancing Fairness in Disease Prediction by Optimizing Multiple Domain Adversarial Networks" to PLOS Digital Health. We appreciate the constructive feedback from the reviewers, which has greatly helped us improve the quality and clarity of our manuscript. We have carefully considered each comment and have revised the manuscript accordingly. Below is a point-by-point response to the reviewers' comments (highlighted in blue), with references to the changes made in the revised manuscript (highlighted also in blue in the revised manuscript).

Reviewer 1:

Comment 1

In the introduction, clarify how MDANN can help mitigate bias.

Response

We have revised the introduction to provide a more explicit explanation of how the MDANN framework mitigates bias. The revised text emphasizes the use of multiple adversarial components to tackle biases in the prediction process and how these adversarial components enable the model to learn representations that are less influenced by sensitive attributes such as sex and handedness. This revision can be found in the Experimental Setup section on pages 2-3 of the manuscript.

Comment 2

In the method, justify why each experiment was run five times.

Response

The revised methods section now includes a justification for running each experiment five times. We clarified that this repetition was performed to account for variability in training due to stochastic processes such as random weight initialization and batch sampling. This ensures that our reported results are robust and generalizable. Running more than five repetitions (e.g., 10 or more) could provide additional insights but comes at the cost of increased computational resources and diminishing returns in terms of variability. Prior research in the field suggests that five repetitions are often sufficient for capturing meaningful trends without requiring extensive computational overhead. To illustrate this point, we ran a set of experiments using 10 repetitions and compared them to those with 5 repetitions. The results were nearly identical, with the average F1 score and variance between 10 and 5 runs showing minimal differences. For example, in the experiment testing MADANN with CAE-2, the F1 score over 5 runs was 0.72 ± 0.01, while over 10 runs, the F1 score was 0.72 ± 0.008. This demonstrates that the additional repetitions did not yield significantly different results, thus we decided to use five repetitions as a sufficient number for capturing meaningful trends without unnecessary computational overhead. Please see page 6 of the Methods section for the updated explanation.

Comment 3

The results show still a low accuracy. Could this be linked to the low sample size?

Response

We agree with the reviewer that sample size could be a contributing factor to the lower accuracy observed in some of our experiments. We have added discussions in the Results section, highlighting how the limited sample size might affect the model's performance and accuracy. Furthermore, we used a synthetic method (e.g., SMOTE) to generate more underrepresentative samples

to enlarge the sample size, which still underperforms MDANN. We have also acknowledged this limitation in the Conclusion and proposed future work to explore larger datasets and additional disease areas. These changes can be found on page 11.

Comment 4

Comment in the conclusion whether this could be transferable to other diseases.

Response

We have expanded the conclusion to address the transferability of the MDANN framework to other diseases. The revised conclusion discusses how the framework's core components—convolutional autoencoders, adversarial components, and AUC-based loss—can be applied to other medical conditions, such as cancer, diabetes, and cardiovascular diseases, where fairness and bias mitigation are also critical. This discussion can be found in the Conclusion on page 11.

Reviewer 2

Comment 1

The feature extractor (CAE) provides data representations but does not guarantee interpretability. Therefore, the quality of the entire system is based on the assumption that the extracted features are correct and interpretable. Could the authors provide more justification?

Response

Thank you so much for your detailed and constructive comments. We have expanded the discussion on the interpretability of the CAE in the methods and results sections. We have revised the manuscript with changes on page 4, page 8 and Table 1.

To address concerns regarding the interpretability and effectiveness of CAE-extracted features, we conducted an ablation study, which is detailed in our manuscript. This study involved systematically removing or altering the CAE module and observing the effects on both predictive performance and fairness metrics across the datasets used (i.e., Autism Brain Imaging Data Exchange II and Alzheimer's Disease Neuroimaging Initiative).

The results from the ablation study clearly demonstrated that the inclusion of CAE significantly enhances the model's ability to mitigate biases and improve overall fairness in disease prediction. For example, as presented in Table 1 of our manuscript, the use of a CAE with two convolutional layers (CAE-2) resulted in the highest accuracy (0.73) and averaged Disparate Impact (DI) of 0.77, compared to lower scores when using original data without CAE-based feature extraction. This indicates that the CAE's deep representations capture critical information that is instrumental in distinguishing between majority and minority groups, thereby contributing to more equitable outcomes.

Furthermore, we acknowledge that the choice of adversarial layers in our Multiple Domain Adversarial Neural Network (MDANN) framework is indeed predicated on the features extracted by the CAE. Our methodology leverages these deep representations to identify and mitigate biases across multiple sensitive attributes, such as age, sex, and handedness. The adversarial components are designed to enforce fairness by learning feature distributions that are invariant to these sensitive attributes, as detailed in the "Adversarial Module" section of our manuscript.

The effectiveness of this approach is empirically validated in our experiments, where the inclusion of adversarial components significantly improved the fairness metrics, as evidenced by higher AUC scores and reduced disparate impact, particularly in scenarios with imbalanced data distributions. This demonstrates that the CAE-extracted features are not only effective in representing

bias features but are also critical for the success of our adversarial training strategy.

However, we acknowledge that while convolutional autoencoders offer powerful deep representations, they may lack interpretability for clinicians. As a result, we have highlighted potential future work in the Discussion section, where we propose incorporating interpretability methods (e.g., attention mechanisms, feature attribution techniques, perturbations) to provide more insights into the model's decisions.

Comment 2

The use of the AUC-based loss function does not inherently account for class imbalance, as traditional sample-weighted loss functions do. Please compare the proposed method with traditional loss functions.

Response

We appreciate the reviewer's thoughtful comments regarding the choice of the AUC-based loss function and its implications for handling class imbalance and fairness. We recognize that class imbalance is a critical issue in biomedical datasets, where minority classes often represent underrepresented or disadvantaged groups, making it essential to ensure that the model does not favor the majority class.

The choice of an AUC-based loss function in our Multiple Domain Adversarial Neural Network (MDANN) framework was motivated by the inherent advantages of the AUC as a performance metric, particularly in scenarios involving class imbalance. Unlike accuracy, which can be misleading in the presence of imbalance, the AUC metric evaluates the model's ability to distinguish between the positive and negative classes across various thresholds, making it less sensitive to skewed class distributions.

In traditional classification tasks, sample-weighted cross-entropy loss is commonly used to address the class imbalance by giving more importance to the minority class during training. However, such an approach may not always align with the goal of maximizing the overall discriminatory power of the model, especially when the dataset is highly imbalanced. The AUC-based loss function, by focusing on the area under the Receiver Operating Characteristic (ROC) curve, provides a more holistic evaluation of the model's performance across different decision thresholds, thereby offering a robust alternative for imbalanced data scenarios.

We clarified that our previous experiment compares the AUC-based minimax loss function with a traditional sample-weighted cross-entropy loss function, particularly for handling class imbalance. In the revised manuscript (page 9), we have complemented that the cross-entropy loss function is sample-weighted, which is not mentioned in the previous manuscript. The results of this comparison, including the performance metrics for both methods, are presented in the Results section on page 9 and 10. As shown in Figure 4, the AUC-based loss function significantly outperforms the sample-weighted cross-entropy loss in terms of both overall classification performance and fairness metrics. Specifically, the AUC scores for models trained with the AUC-based loss function were consistently higher across different sensitive attributes, indicating better discriminatory power and more balanced performance across majority and minority groups.

Moreover, the accuracy for the minority class improved when using the AUC-based loss function, particularly in highly imbalanced settings. For example, the accuracy for left-handed individuals in the ADNI dataset, who represent a minority group, increased from 0.6 with cross-entropy loss to 0.8 with the AUC-based loss. This improvement highlights the effectiveness of the AUC-based loss function in addressing the class imbalance issue while maintaining high predictive per-

formance.

Comment 3

Finally, the use of deep learning techniques, such as convolutional autoencoders (CAEs) and adversarial networks, may reduce the interpretability of the model, making it harder for clinicians to understand and trust the predictions. While these advanced techniques can enhance prediction accuracy and fairness, their complex nature can obscure how specific features contribute to the model's decisions. This lack of transparency can be a significant barrier to the adoption of such models in clinical practice, where understanding the rationale behind predictions is crucial for informed decision-making and patient trust.

Response

We acknowledge the reviewer's valid concern regarding the interpretability of deep learning models, particularly in the context of clinical applications. While convolutional autoencoders (CAEs) and adversarial networks have demonstrated improvements in prediction accuracy and fairness, we agree that their complexity may reduce transparency, making it difficult for clinicians to trust and understand the predictions.

In response to this concern, we have expanded the Discussion section to propose ways to improve the interpretability of our MDANN framework. We suggest incorporating feature attribution methods (e.g., Grad-CAM, LIME) and attention mechanisms in future work, which can help highlight the specific parts of the input data that influence the model's decisions. These techniques can provide clinicians with more insights into the model's reasoning and aid in building trust. Additionally, we propose further testing of MDANN alongside simpler, interpretable models such as logistic regression or decision trees to provide comparison baselines that clinicians can more easily understand.

Moreover, to address concerns about model complexity and transparency, we included a comparison with the traditional method SMOTE in the revised manuscript. SMOTE is a well-known technique that addresses class imbalance and is commonly used due to its simplicity and interpretability. By comparing SMOTE with MDANN, we demonstrate that while SMOTE improves fairness in predictions, it falls short in terms of accuracy and overall fairness metrics compared to MDANN. This comparison underscores the trade-off between interpretability and performance. The inclusion of SMOTE in the results highlights that traditional methods, while simpler, may not achieve the same level of fairness and accuracy as MDANN, particularly when addressing multiple sensitive attributes. These results are now included in the revised Results section and presented in Figure D. These updates can be found on pages 10-11 of the revised manuscript.

Comment 4

The acronym CAE is not defined in the introduction.

Response

Thank you for your reminder. The acronym CAE (Convolutional Autoencoder) is now defined in the introduction on page 3.

Comment 5

The definitions of "majority" and "minority" are missing in Equations 2 and 3, which may lead to ambiguity in interpreting the equations.

Response
We have revised the Evaluation Metrics section on page 7 to explicitly define "majority" and "minority" groups in the context of our study. These definitions are now clearly stated, and the corresponding equations have been adjusted to avoid any ambiguity.

Comment 6
Table 1 should report the F1-score instead of accuracy. When dealing with imbalanced datasets, accuracy is not a reliable metric, and the F1-score provides a better measure of the model's performance.

Response
Thank you for your suggestion. We have replaced accuracy with F1-score in Table 1 to better reflect the model's performance on imbalanced datasets. The revised table can be found on page 9.

Comment 7
The quality of the figures should be improved, for example by using PDF format or higher resolution images to ensure clarity and readability.
Response
Thank you for your comments. We have updated all figures to a higher resolution for clarity and readability.
