# Supplementary material for: Enhancing fairness in disease prediction by optimizing multiple domain adversarial networks
Source: PLOS Digit Health. 2025 May 30;4(5):e0000830. doi: 10.1371/journal.pdig.0000830 (PMC12124548; doi:10.1371/journal.pdig.0000830)
Supplement: Attachment — Submitted filename: Response to Reviewers.pdf [file pdig.0000830.s004.pdf]

Dear Editors,

Thank you for giving us the opportunity to submit a revised version of our manuscript titled "Enhancing Fairness in Disease Prediction by Optimizing Multiple Domain Adversarial Networks" to PLOS Digital Health. We appreciate the constructive feedback from the reviewers, which has greatly helped us improve the quality and clarity of our manuscript. We have carefully considered each comment and have revised the manuscript accordingly. Below is a point-by-point response to the reviewers' comments (highlighted in blue), with references to the changes made in the revised manuscript (highlighted also in blue in the revised manuscript).

Reviewer 2:

Comment 1

The fairness component is not rigorously addressed. The use of convolutional autoencoders (CAEs) to extract "deep representations" is problematic because CAEs are designed to preserve the essential components of the data, which may include biased features.

Response

Thank you for raising this concern. To address this issue, we have conducted additional experiments to evaluate how "deep representations" learned by CAEs disentangle biased features. We analyzed the correlations between sensitive attributes (e.g., demographic traits such as sex and handedness) and the extracted embeddings. Specifically, the correlations were computed using the Pearson correlation coefficient, which quantifies the linear relationship between sensitive attributes (binary values) and embedding features. The computation process is as follows:

- Embedding Feature Aggregation: For each individual, embeddings generated by the CAE modules (CAE-1, CAE-2, and CAE-3) or raw features (Baseline without CAE) were aggregated across dimensions. This produced a single scalar value per individual, representing the overall embedding.
- Attribute-Feature Mapping: Sensitive attributes (e.g., sex or handedness) were mapped as binary values (e.g., 0 for male and 1 for female) to each individual.
- Correlation Computation: Using the aggregated embedding values and binary-sensitive attributes, the Pearson correlation coefficient was calculated to measure the strength and direction of the relationship.

These results, added to the Section Result and Table 2, demonstrate that embeddings generated by the CAE combined with adversarial modules reduce sensitive feature correlations.

Table 1: **Performance of MDANN with different CAE modules.**

Module	Embedding Size	Convolutional layers	FID score	F1 score	Averaged DI
N/A	N/A	N/A	N/A	0.61	0.68
CAE-1	64×64×128	1	556.74	0.61	0.67
**CAE-2**	**32×32×256**	**2**	**490.98**	**0.72**	**0.77**
CAE-3	16×16×512	3	555.54	0.67	0.72

Comment 2

The manuscript employs AUC-based loss without sufficient justification. A comparison with weighted cross-entropy loss is missing.

Table 2: **Correlation analysis between sensitive attributes and embeddings across different CAE modules.**

Module	Correlation with Sex	Correlation with Handedness
Baseline (No CAE)	0.45	0.52
CAE-1	0.38	0.45
**CAE-2**	**0.20**	**0.18**
CAE-3	0.25	0.22

Response

We conducted a comparative analysis between AUC-induced minimax loss and weighted cross-entropy loss.

The results, added to Section and Figure 4, show that the minimax loss consistently outperforms weighted cross-entropy in terms of both classification performance (AUC score) and fairness metrics (DI and EO). For example, on the ADNI dataset, the AUC-induced loss improved DI by 18% compared to the weighted cross-entropy loss. This result underscores the superiority of AUC-based optimization in handling class imbalance and fairness.

To further justify the choice of AUC-based loss, we added the following key points to the manuscript (Section 3.2):

- Threshold Independence: Unlike cross-entropy loss, AUC-based optimization does not rely on a fixed decision threshold, which allows the model to optimize over the entire range of operating points. This property is particularly valuable in biomedical datasets, where the optimal threshold may vary due to class imbalance and fairness considerations.

- Robustness to Class Imbalance: By aggregating performance across all thresholds, the AUC-based loss inherently balances the model's attention between majority and minority classes, preventing the dominance of one over the other. This approach is especially critical for datasets like ADNI, where the majority-to-minority ratio exceeds 2:1 for some sensitive attributes.

- Fairness Trade-offs: Optimizing AUC directly influences sensitivity (True Positive Rate) and specificity (True Negative Rate), integral to fairness metrics such as EO and TNR disparity. Weighted cross-entropy loss, while effective for adjusting class importance, does not explicitly account for these trade-offs, making it less suitable for scenarios requiring fairness enhancements.

Comment 3

Accuracy is inappropriate for imbalanced and biased datasets. Metrics like F1-score, precision, recall, or balanced accuracy should be included.

Response

We agree that accuracy alone is insufficient for imbalanced datasets. We have thus included more metrics in the Results.

- We included F1-scores in Table 1. Unlike accuracy, which can be misleading in imbalanced datasets, F1-scores account for both false positives and false negatives, emphasizing the

model's performance on underrepresented groups. This makes F1-scores a more reliable measure in scenarios with imbalanced data. In addition, We add a new Table 3, which describes the reported Accuracy (ACC), AUC score, Disparate Impact (DI), Equal Opportunity (EO), and True Negative Rate Disparity (TNRD) to provide a detailed evaluation of the fairness metrics across different sensitive attributes. Thus, Table 3 provides a comprehensive evaluation of fairness metrics (DI, EO, TNRD) alongside classification metrics (ACC, AUC), explicitly assessing how well the model performs across different sensitive attributes.

- In the Results section, we discussed how these metrics capture the model's performance on minority classes, highlighting improvements in fairness and classification outcomes.

Comment 4

Figure formatting issues hinder readability.

Response

We have addressed formatting issues: The caption of Figure 1 has been corrected to align properly with the figure. Figures 3 and 5 have been resized and relabeled to improve clarity. The caption of Figure 6 has been revised to specify the task reported in the figure.

Reviewer 3

Comment 1

Details about data partitioning or cross-validation are missing.

Response

We expanded the Section Dataset to include specific details about our cross-validation procedure:

A 5-fold stratified cross-validation method was used to ensure a balanced representation of classes and sensitive attributes in training and validation splits. We provided specific ratios for training (80%) and testing (20%), stratified by sensitive features like sex and handedness. These details ensure reproducibility and clarify the robustness of our evaluation process.

Comment 2

On the equations of fairness metrics: a) Y is not defined in the manuscript. In the context of this study, Y the ground truths, or the model predictions? b) "the proportion of the minority group that received 231 the positive outcome": what is the "positive outcome" specifically in detecting Alzheimer's Disease and Autism? c) The equation for EO is incorrect. In order to estimate true positive rate, both ground truth and model prediction should be present in this equation. However only "Y" was included (which is also not clear whether it denotes the ground truths of the predictions). d) For disparity in recalls, the authors only reported the disparity in true positive rate (TPR). However, only reporting TPR disparity does not capture the whole picture of the fairness performance trade-off (an extreme example being both demographics group both having TPRs of 0, which is still considered perfectly fair if only disparity for TPR were reported). For a more comprehansive evaluation, please report the disparity of true negative rate, and the performance metrics that which the fairness metrics are derived from (DI: selection rate; EO: TPR; TNR disparity: TNR)

Response

a). We revised the manuscript to clarify fairness metric equations. Y now explicitly denotes the ground truth labels, while model predictions are denoted as $\hat{Y}$.

b). The manuscript now clearly specifies what constitutes a "positive outcome" for the classification tasks:

For Alzheimer's Disease (AD), the positive outcome refers to a prediction of mild cognitive impairment (MCI) or AD, as opposed to normal cognition (NC). For Autism Spectrum Disorder (ASD), the positive outcome refers to a prediction of ASD as opposed to typical controls (TC). These definitions have been added to the section where fairness metrics are introduced.

c). We have revised the equation for Equal Opportunity (EO) to accurately reflect the true positive rate (TPR) disparity. The updated equation is:

$$\begin{aligned} EO &= |TPR_{minority} - TPR_{majority}| \\ &= |Pr(\hat{Y} = 1|Y = 1, D = minority) - Pr(\hat{Y} = 1|Y = 1, D = majority)| \end{aligned} \quad (1)$$

This equation now accounts for both ground truths (Y) and predictions ($\hat{Y}$), ensuring that TPR disparity is correctly defined. This updated equation have been included in the revised manuscript for clarity.

These changes make the equations clearer and align with standard definitions in fairness literature.

d). To provide a more comprehensive evaluation of fairness, we now include true negative rate disparity (TNRD) alongside EO in Table 3. TNRD is calculated as:

$$\begin{aligned} TNRD &= |TNR_{minority} - TNR_{majority}| \\ &= |Pr(\hat{Y} = 0|Y = 0, D = minority) - Pr(\hat{Y} = 0|Y = 0, D = majority)| \end{aligned} \quad (2)$$

e). Following the reviewer's suggestion, we now explicitly report the performance metrics underpinning fairness metrics, including DI:

$$DI = \frac{Sr_{minority}}{Sr_{majority}}, SR = Pr(\hat{Y} = 1|D = Group) \quad (3)$$

Comment 3

Descriptions of Figure 5 panels are inconsistent with the captions.

Response

We revised the manuscript to ensure the descriptions of Figure 5 panels are consistent with the figure captions. Each panel is now explicitly referenced and described in the text.

Comment 4

For the performance metrics and fairness metrics reported in this manuscript, please also report their variation (e.g. standard deviation) or associated estimates of uncertainty (e.g. confidence intervals).

Response

Thank you for your invaluable suggestion regarding the inclusion of variation estimates for the performance and fairness metrics. In response, we have updated the manuscript to report standard deviations for all metrics in Table 3, calculated based on repeated cross-validation (five folds) across the dataset.

Comment 5

Caption for Fig. 6: For better clarity, please specify what classification task is used in this figure. Same for the captions of other figures & tables.

Response

Thank you for pointing out the need for additional clarity in the captions of figures and tables. We have revised the captions throughout the manuscript to state the classification task being discussed.

Comment 6

On the description for Fig. 6: EO was not reported in the main text. Please report all performance/fairness metrics described in this study in either main text, tables or supplementary tables, for each of the tasks, and models compared.

Response

Thank you for highlighting the need to comprehensively report EO alongside other performance and fairness metrics in the main text. To address this concern, we have revised the main text to explicitly report EO for both sensitive features (sex and handedness) in Autism classification tasks. The revised text now discusses EO values alongside Accuracy (ACC), AUC, and Disparate Impact (DI) for each of the models and methods compared.

Comment 7

On subsection "Bias mitigation via multiple components in the adversarial module": It comes to my attention that no fairness metric was reported for AD prediction in this subsection (Fig. 5 only includes performance metrics). As a result, there is no quantitative evidence that draws to the conclusion that "fairness was significantly improved without sacrificing classification performance". Same the previous comment, please provide comprehensive quantitative results for both of the classification tasks.

Response

We agree that including fairness metrics is crucial to substantiate the claim that fairness was significantly improved without sacrificing classification performance.

To address this concern, we have revised the subsection and introduced Table 3 to include comprehensive quantitative results for both performance and fairness metrics (DI and EO) for the AD prediction task. Specifically, the revised text now reports DI and EO for each sensitive feature (age, education years, and handedness) alongside accuracy and AUC under different configurations (Baseline, MDANN with 1 AC, 2 ACs, and 3 ACs). These results demonstrate the improvements in fairness metrics achieved by increasing the number of adversarial components, as well as the trade-offs in predictive performance.

Comment 8

The authors mentioned in the introduction that "biases can intersect in complex ways that are difficult to disentangle and address with single-attribute-focused methods". However, it should be noted that the methodology and evaluation in this study is still essentially based on marginal fairness, i.e. fairness defined by single sensitive attributes (although their model can mitigate biases for each of the attributes simultaneously, the adversarial modules still operate independently without considering the interactions between sensitive attributes). Although marginal fairness could be used to approximate intersectional fairness, there is still a distinction between these two fairness

Table 3: **Fairness Metrics with Standard Deviations Across Sensitive Attributes (A_1: Age, A_2: Educated Years, A_3: Handedness) with Varying Numbers of Adversarial Components (ACs).**

Configuration	ACC	AUC	DI			EO		
			A_1	A_2	A_3	A_1	A_2	A_3
Baseline	0.69 ±0.02	0.71 ±0.02	0.61 ±0.04	0.60 ±0.05	0.63 ±0.04	0.19 ±0.03	0.21 ±0.04	0.19 ±0.03
1 AC	0.74 ±0.03	0.77 ±0.03	0.65 ±0.03	0.68 ±0.04	0.68 ±0.03	0.15 ±0.02	0.16 ±0.03	0.14 ±0.02
2 ACs	0.78 ±0.02	0.81 ±0.02	0.71 ±0.02	0.72 ±0.03	0.73 ±0.02	0.10 ±0.01	0.11 ±0.02	0.09 ±0.01
3 ACs	0.83 ±0.01	0.85 ±0.01	0.76 ±0.02	0.77 ±0.02	0.78 ±0.02	0.05 ±0.01	0.06 ±0.01	0.04 ±0.01

definitions, and marginal fairness on all individual attributes does not guarantee fairness in intersectional subgroups defined by multiple attributes. That being said, it would be beneficial if the author could also evaluate fairness across intersectional subgroups defined by multiple attributes (if it is feasible for the sample sizes in this study). Or, in the very least, the authors should clarify that their method assumes no bias interaction between sensitive attributes.

Response

We thank the reviewer for raising this important point regarding the distinction between marginal fairness and intersectional fairness. We acknowledge that while the MDANN framework mitigates biases for multiple sensitive attributes simultaneously, the adversarial modules currently operate independently and do not explicitly account for potential interactions between these attributes. As such, the fairness evaluation in this study focuses on marginal fairness, where each sensitive attribute is considered independently.

To address the reviewer's comment, we have revised the Conclusion section to explicitly acknowledge this limitation. We clarify that the current framework assumes no significant bias interaction between sensitive attributes, simplifying the fairness evaluation to the level of individual attributes. Additionally, we discuss the distinction between marginal and intersectional fairness, noting that marginal fairness does not guarantee fairness for subgroups defined by combinations of sensitive attributes.

Given the limited sample sizes for certain intersectional subgroups in the ABIDE II and ADNI datasets, a robust evaluation of intersectional fairness was not feasible in this study. However, we propose this as an important direction for future work. Specifically, we suggest extending the MDANN framework to incorporate subgroup-specific adversarial components or hierarchical modeling approaches to explicitly address intersectional fairness. Such advancements would enable the framework to capture and mitigate the complex interactions between sensitive attributes, enhancing fairness across overlapping demographic subgroups.

We believe these revisions clarify the scope of our methodology and provide transparency about its limitations, while also outlining concrete directions for future research to address intersectional fairness. We hope this addresses the reviewer's concerns thoroughly.

Comment 9

Fig. 3 only provide a partial view of the demographic distribution, since we cannot tell if there is any

difference in class distribution between patient groups. Please consider also report class-stratified demographics. In addition, please also provide specific patient counts in addition to percentages.

Response

We reported a new supplementary table (Supplementary Table 1 and Table 2). This table provides detailed counts and percentages for each demographic group within each class.

Table 4: **Class-Stratified Demographics for the ADNI Dataset Across Sensitive Attributes.**

Class	Age Groups		Educated Years		Handedness	
	<78	≥78	<16 years	≥16 years	Right-Handed	Left-Handed
NC	230 (65.7%)	120 (34.3%)	206 (58.9%)	144 (41.1%)	340 (97.1%)	10 (2.9%)
MCI	107 (70.4%)	45 (29.6%)	125 (82.2%)	27 (17.8%)	130 (85.5%)	22 (14.5%)
AD	113 (67.3%)	55 (32.7%)	139 (82.7%)	29 (17.3%)	150 (89.3%)	18 (10.7%)

Table 5: **Class-Stratified Demographics for the Autism Dataset Across Sensitive Attributes.**

Class	Sex		Handedness	
	Male	Female	Right-Handed	Left-Handed
ASD	56 (50.9%)	54 (49.1%)	104 (94.5%)	6 (5.5%)
TC	86 (84.3%)	16 (15.7%)	86 (84.3%)	16 (15.7%)
