# Supplementary material for: Enhancing fairness in disease prediction by optimizing multiple domain adversarial networks
Source: PLOS Digit Health. 2025 May 30;4(5):e0000830. doi: 10.1371/journal.pdig.0000830 (PMC12124548; doi:10.1371/journal.pdig.0000830)
Supplement: Attachment — Submitted filename: Response_to_Reviewers_auresp_3.pdf [file pdig.0000830.s005.pdf]

Dear Editors,

Thank you for giving us the opportunity to submit a revised version of our manuscript titled "Enhancing Fairness in Disease Prediction by Optimizing Multiple Domain Adversarial Networks" to PLOS Digital Health. We appreciate the constructive feedback from the reviewers, which has greatly helped us improve the quality and clarity of our manuscript. We have carefully considered each comment and have revised the manuscript accordingly. Below is a point-by-point response to the reviewers' comments (highlighted in blue), with references to the changes made in the revised manuscript (highlighted also in blue in the revised manuscript).

Reviewer 3:

Comment 1

The authors claimed to have added TNRD to the manuscript. But I could not find any specific value of TNRD throughout their revised manuscript.

Response

Thank you for raising this concern. We acknowledge the oversight and have now explicitly included the specific values of True Negative Rate Disparity (TNRD) in the revised manuscript. These values are reported alongside Disparate Impact (DI) and Equal Opportunity Difference (EOD) in Table 3 for a comprehensive fairness evaluation. Additionally, we have updated the Results section to explicitly discuss TNRD and its role in evaluating model fairness, ensuring that all fairness metrics are consistently reported.

Comment 2

The authors do not seem to have fully addressed these comments. They only included F1-score in Table 1 (which is only an ablation study for CAEs), while the performance metrics mentioned above are still missing from the rest of the manuscript. The only two performance metrics that were consistently reported throughout the manuscript are accuracy and AUROC, which - as mentioned in the previous comments - do not fully capture the model performance and fairness-performance trade-off.

Response

We appreciate this important observation and have taken the following steps to address the concern:

- Expanded Performance Metrics Beyond Accuracy and AUC: In addition to accuracy and AUC, we have also reported F1-score for a more comprehensive analysis of model performance across imbalanced datasets. F1-scores provide deeper insights into how well our model balances sensitivity and specificity, particularly for minority class predictions.

- Revised Tables and Figures: We have revised Table 3 to include F1-score and standard deviations for all configurations. Additionally, F1 and TNRD have been provided in the revised Figure 6 to demonstrate how different methods perform across both classification and fairness metrics, further clarifying trade-offs between performance and bias mitigation.
